# Perspectives in clinical microbiology for combating multi-drug resistant bacterial infections

**DOI:** 10.3389/fcimb.2025.1695284

**Published:** 2025-09-23

**Authors:** André Mauricio de Oliveira, Camila Prósperi de Castro

**Affiliations:** Department of Environment, Federal Centre of Technological Education of Minas Gerais, Contagem, Brazil

**Keywords:** multidrug-resistant bacterial infections, broad-spectrum antibiotics, microbiota, bacteriophages, microbiome

## Abstract

Multidrug-resistant bacterial infections are a major global threat, exacerbated by globalization and poor sanitation. Bacteria develop resistance through mechanisms like enzymatic degradation, efflux pumps, and horizontal gene transfer. Rapid diagnostics and artificial intelligence are crucial for overcoming the limitations of traditional culture methods. Combating this issue requires novel therapeutic strategies, such as bacteriophages, antimicrobial peptides, and microbiome-based therapies. Ultimately, proper antibiotic use, increased research, and global multidisciplinary cooperation are essential to address this complex challenge.

## 1 Introduction

Multidrug-resistant bacterial (MDR) infections are a threat in a globalized world. They are caused by bacteria that have developed resistance to multiple antimicrobial agents, particularly those commonly used for treatment. MDR bacteria are characterized by non-susceptibility to at least one agent in three or more antimicrobial categories, limiting therapeutic options and increasing the risk of treatment failure, prolonged illness, and higher mortality rates (Magiorakos et al., 2012). Some well-known MDR pathogens include methicillin-resistant *Staphylococcus aureus* (MRSA), extended-spectrum β-lactamase (ESBL)-producing *Enterobacteriaceae*, carbapenem-resistant *Pseudomonas aeruginosa*, and *Acinetobacter baumannii* (Antibiotic resistance threats in the United States, 2019). The emergence of antibiotic-resistant bacteria is a relatively recent phenomenon, catalyzed by population growth, increased international trade, with consequent growth in the use of air transport (79th Annual General Meeting and World Air Transport, 2023) and poor sanitary conditions in megacities, the majority of which are located on the Asian continent (Hawkey, 2015). Most Gram-negative bacteria carried by passenger are *Escherichia coli* and *Klebsiella* spp. that belong to *Enterobacteriaceae* family and are known to have a more fluid genome, presenting antibiotic resistance genes. Intercontinental flights accelerated the spread of COVID-19, H1N1 and Ebola, with initial outbreaks linked to hubs such as Wuhan, Milan, and New York (Bogoch et al., 2020). Individual initiatives and global public health strategies, including vaccination, can contain the spread of bacterial resistance (Jansen et al., 2021).

## 2 Mechanisms of antibiotic resistance

The development of antibiotic resistance is a multifaceted biological process shaped by environmental factors, the density of microbial populations, and the extensive use of antibiotics in medicine, farming, and the food industry (Jansen et al., 2021). When first exposed to a new antibiotic, bacteria typically exhibit high susceptibility and elevated mortality rates. However, a few rare individuals survive—usually due to genetic mutations that provide adaptive advantages. These mutations are passed down to daughter cells during reproduction. Given the rapid reproduction rate of bacteria, this process enables nearly the entire population to become resistant to the antibiotic in a very short period. Additionally, bacteria can spread antibiotic resistance through horizontal gene transfer processes such as conjugation (direct cell-to-cell transfer via plasmids), transduction (virus-mediated transfer), or transformation (uptake of free DNA) (Hawkey, 2015; Tortora et al., 2004).

### 2.1 How do bacteria develop resistance

**Figure 1:**
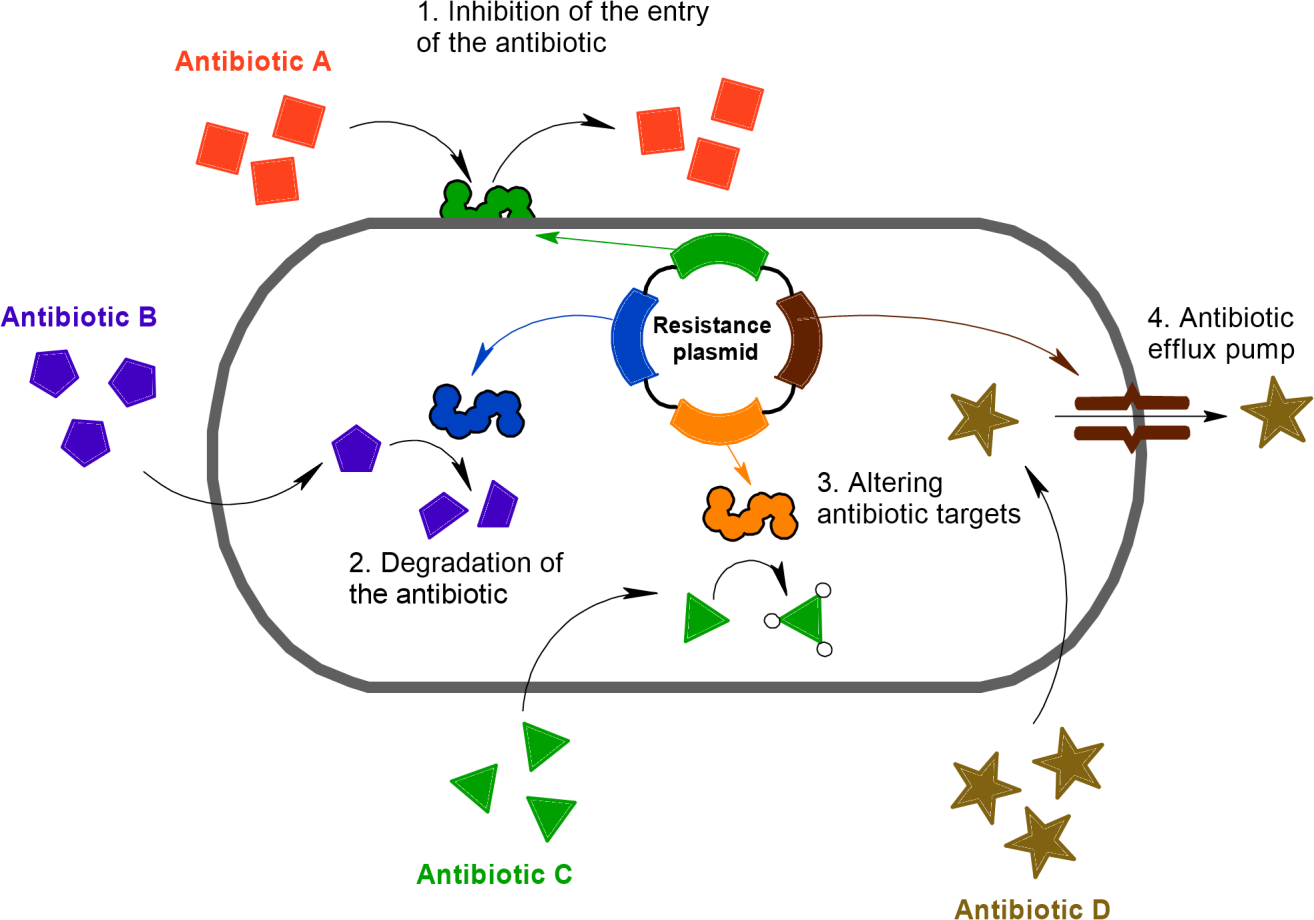
Main mechanisms of bacterial resistance to antibiotics: (1) inhibition of drug entry through reduced membrane permeability, (2) enzymatic degradation of the antibiotic, (3) modification of antibiotic targets to reduce binding affinity, and (4) active efflux of the drug via efflux pumps. These resistance traits are often encoded by genes located on plasmids, which can be horizontally transferred between bacterial cells, contributing to the spread of antibiotic resistance. Source: author´s content.

Bacteria can develop resistance to antibiotics through multiple mechanisms. One common strategy involves modifications in the cell wall or membrane, which block the antibiotic from entering the bacterial cell. Another well-known mechanism is the production of enzymes that degrade the antibiotic, rendering it inactive and unable to eliminate the infection. A third mechanism consists of structural changes in the bacterial cell that alter the usual targets of antibiotics, thereby reducing their effectiveness. Additionally, some bacteria use efflux pumps — molecular “safety valves” that actively expel antibiotics from the cell before they can act. (**Figure 1**) (Kakoullis et al., 2021). An MDR bacterium can — and often does — exhibit multiple antibiotic resistance mechanisms simultaneously, making it particularly difficult to treat with standard antimicrobial therapies.

## 3 Challenges in diagnosing MDR infections

### 3.1 A rapid and accurate diagnosis is required for effective treatment, but traditional diagnostic methods have limitations

The rapid and accurate identification of multidrug-resistant (MDR) bacteria is crucial for initiating appropriate and effective therapies. Traditional diagnostic methods, such as microbiological culture followed by biochemical and susceptibility testing, remain widely used but have important limitations. In addition to the time required, conventional methods struggle with the early detection of resistance, particularly in infections caused by bacteria with complex resistance mechanisms. Another significant drawback is that these techniques only identify bacteria capable of growing under laboratory conditions, which means they miss non-cultivable or slow-growing microorganisms that may carry antimicrobial resistance (AMR) genes (Ahmad et al., 2024; Galhano et al., 2021; Ramzan et al., 2024). These limitations can delay the initiation of effective antimicrobial therapy, often leading to the empirical use of broad-spectrum antibiotics, which further contributes to the rise of resistance among bacteria. Given the limitations of traditional techniques, there is a growing demand for faster and more sensitive diagnostic methods, such as Polymerase Chain Reaction (PCR), Mass Spectrometry (MALDI-TOF MS), and Next-Generation Sequencing (NGS). These molecular approaches are highly effective in identifying pathogens and their resistance genes directly from clinical samples, often within just a few hours (Ahmad et al., 2024), in reducing the time required for diagnosis, and in the improvement of the accuracy of antimicrobial resistance detection, supporting a more rational and targeted use of antibiotics. As a result, these methods represent a promising advancement in the surveillance and control of infections caused by MDR bacteria.

### 3.2 Artificial intelligence and automation improve diagnostic speed and accuracy, overcoming the limitations of traditional techniques

Artificial intelligence (AI) computational methods are able to mimic natural human decisions, and science has taken advantage of it to deal with complex and multicomponent issues. As expected, AI-based strategies have drawn new ways for combating diseases as feasible alternatives to traditional trial-and-error approaches, and some AI tools that have been proven to be useful for this sake (Ndikuryayo et al., 2025). Some are oriented to small molecules design, mainly for peptide-based antibiotics design, such as AMPlify (Li et al., 2022), AI4AMP (Lin et al., 2021), Macrel (Santos-Júnior et al., 2020) and CalcAMP (Bournez et al., 2023); some are focused on the identification of antibiotic resistance genes, such as VAMPr (Kim et al., 2020), CARD 2023 (Alcock et al., 2023), HMDARG (Li et al., 2021), ARG‐SHINE (Wang et al., 2021). There are some developed to specific diseases, such as GenTB, for tuberculosis (Gröschel et al., 2021). An interesting case that illustrates how AI enables the development of new drugs against MDR is the discovery of halicin in 2020 as a potential compound for this purpose (Stokes et al., 2020). The study began with a search using an AI strategy called deep neural network (DNN) against a database of over 100 million compounds capable of inhibiting the growth of *Escherichia coli*. After selecting halicin, *in vitro* tests demonstrated its efficacy against a range of other resistant bacteria, such as *Mycobacterium tuberculosis*, *Enterobacteriaceae* sp., *Clostridioides difficile*, and *Acinetobacter baumannii* (making it the WHO’s drug of choice against this one). Given that this computational effort depends on an up-to-date database of resistant bacteria, it is always necessary to be aware of these resources (**Table 1**).

**Table 1:** Web databases of multiresistant bacteria.

Database	Description	Reference
WHO Bacterial Priority Pathogens List (BPPL)	This list from the World Health Organization (WHO) categorizes bacteria based on their threat level and guides research and development efforts	(WHO, 2024)
National Database of Antibiotic Resistant Organisms (NDARO)	This U.S. National Institutes of Health (NIH) database tracks antimicrobial resistance in pathogens	(NCBI, 2025)
Antimicrobial Resistance (AMR) Surveillance in the United States	The U.S. Centers for Disease Control and Prevention (CDC) monitors antimicrobial resistance threats.	(Centers for Disease Control and Prevention, 2019)
University of São Paulo’s database	This open-access database focuses on microorganisms classified as critical priorities by the WHO	(Fuga et al., 2022)
The Comprehensive Antibiotic Resistance Database (CARD)	A bioinformatics resource that provides data on antibiotic resistance genes, mechanisms, and associated pathogens.	(Alcock et al., 2023)
ATLAS (Antimicrobial Testing Leadership and Surveillance)	A database by Pfizer (now part of IHMA) that tracks global antibiotic resistance patterns.	(ECDC, 2025)

## 4 Search for new therapeutic strategies

### 4.1 Research for new antibiotics, the use of bacteriophages, antimicrobial peptides, and microbiome-based approaches

In recent years, alternative approaches to antibiotics have attracted significant interest due to their distinct mechanisms of action compared to traditional antimicrobial drugs. These innovative strategies hold the potential to fundamentally change how MDR infections are treated (Murugaiyan et al., 2022). One area of growing interest involves the human microbiota, whose complex network of microorganisms plays a vital role in protecting the host against pathogens. Through competitive exclusion, commensal bacteria limit pathogenic microbes’ access to essential resources, effectively suppressing their growth. Moreover, research into the human microbiome has uncovered new antimicrobial compounds, highlighting microbiome-based therapies as an emerging and promising approach to combat MDR infections, despite being relatively underexplored (Nhu and Young, 2023).

Another compelling alternative is the use of bacteriophages—viruses that infect and kill bacterial pathogens. Their high specificity for target bacteria makes bacteriophages a powerful tool in the fight against infections caused by MDR microorganisms (Kwiatek et al., 2020). A well-known reported case of this strategiy is the successful treatment of a disseminated *Mycobacterium abscessus* infection in a patient with cystic fibrosis (Dedrick et al., 2019).

Furthermore, natural plant compounds are a promising group of antimicrobial agents. Polyphenols such as tannins and catechins work against a wide range of microbes by breaking down bacterial cell walls, attaching to proteins, and disrupting essential metabolic processes (Elkhalifa et al., 2024; Kwiatek et al., 2020). Taken together, these alternative strategies offer promising avenues to address the escalating challenge of multidrug-resistant infections. While each approach has its own strengths and limitations, continued research and integration of these methods could lead to more effective and sustainable treatments, ultimately reducing our reliance on traditional antibiotics and helping curb the spread of resistance.

### 4.2 The potential of personalized medicine in combating MDR infections

It is well known that genetic differences influence drug efficacy, given that small changes in the expression of biological receptors can translate into different antibiotic affinities (Zhou et al., 2021). Why not leverage this characteristic to seek personalized antibiotics capable of inhibiting the growth of pathogens according to each individual’s biochemical specificity? Naturally, the challenge is to design specific drugs capable of responding to subtle structural differences governed by genetic factors, even considering all known drug design strategies. This field has been called pharmacogenomics, and has been used, for example, to study G-protein-coupled receptors (GPCRs) as drug targets (Hauser et al., 2018). One class of antibiotic candidates that allows for enormous structural diversity is oligopeptides.

## 5 Conclusion

The proper use of antibiotics, following the prescribed dose, treatment duration, and correct administration times, is essential for the therapeutic effectiveness. Indiscriminate use of these drugs significantly contributes to the selection and spread of resistant strains, representing one of the main current challenges in combating infections caused by MDR bacteria. The administration of antibiotics disrupts the native microbiota of the host, selecting for resistant bacterial strains that may subsequently cause opportunistic infections (Dongre et al., 2025). The role of science in fighting disease and extending life expectancy is a process that feeds back on the adaptations that pathogens undergo as the population grows and diversifies genetically. Clinical microbiology exceeds a critical role in combating MDR bacteria, with its methods and strategies. However, as a counterpart to the resources that science provides in this matter, actions by national governments are important so that scientific advances have adequate and efficient applicability. Increased awareness, research, and global cooperation is called to action. The complexity of the problem requires multiple knowledge and good communication between professionals on a multi and interdisciplinary basis.

## References

[B1] 79th Annual General Meeting and World Air Transport (2023). International Air Transport Association Annual Review 2023. Available online at: https://www.icao.int/sustainability/WorldofAirTransport/Pages/the-world-of-air-transport-in-2023.aspx (Accessed September 13, 2025).

[B2] Ahmad S. Lohiya S. Taksande A. Meshram R. J. Varma A. Vagha K. (2024). A comprehensive review of innovative paradigms in microbial detection and antimicrobial resistance: beyond traditional cultural methods. Cureus 16. doi: 10.7759/CUREUS.61476, PMID: 38952583 PMC11216122

[B3] Alcock B. P. Huynh W. Chalil R. Smith K. W. Raphenya A. R. Wlodarski M. A.. (2023). CARD 2023: expanded curation, support for machine learning, and resistome prediction at the Comprehensive Antibiotic Resistance Database. Nucleic Acids Res. 51, D690–D699. doi: 10.1093/NAR/GKAC920, PMID: 36263822 PMC9825576

[B4] Bogoch I. I. Watts A. Thomas-Bachli A. Huber C. Kraemer M. U. G. Khan K. (2020). Pneumonia of unknown aetiology in Wuhan, China: potential for international spread via commercial air travel. J. Travel Med. 27, 1–3. doi: 10.1093/JTM/TAAA008, PMID: 31943059 PMC7107534

[B5] Bournez C. Riool M. de Boer L. Cordfunke R. A. de Best L. van Leeuwen R.. (2023). CalcAMP: A new machine learning model for the accurate prediction of antimicrobial activity of peptides. Antibiotics 12, 1–21. doi: 10.3390/ANTIBIOTICS12040725, PMID: 37107088 PMC10135148

[B6] Centers for Disease Control and Prevention (U.S.); National Center for Emerging Zoonotic and Infectious Diseases (U.S.). Division of Healthcare Quality Promotion. (2019). Antibiotic resistance threats in the United States. Atlanta, Georgia: CDC. City. doi: 10.15620/CDC:82532

[B7] Dedrick R. M. Guerrero-Bustamante C. A. Garlena R. A. Russell D. A. Ford K. Harris K.. (2019). Engineered bacteriophages for treatment of a patient with a disseminated drug-resistant Mycobacterium abscessus. Nat. Med. 25, 730–733. doi: 10.1038/s41591-019-0437-z, PMID: 31068712 PMC6557439

[B8] Dongre D. S. Saha U. B. Saroj S. D. (2025). Exploring the role of gut microbiota in antibiotic resistance and prevention. Ann. Med. 57, 2478317. doi: 10.1080/07853890.2025.2478317, PMID: 40096354 PMC11915737

[B9] ECDC (2025). Surveillance Atlas of Infectious Diseases. Available online at: https://atlas.ecdc.europa.eu/public/index.aspx.

[B10] Elkhalifa M. E. M. Ashraf M. Ahmed A. Usman A. Hamdoon A. A. E. Elawad M. A.. (2024). Polyphenols and their nanoformulations as potential antibiofilm agents against multidrug-resistant pathogens. Future Microbiol. 19, 255–279. doi: 10.2217/FMB-2023-0175, PMID: 38305223

[B11] Fuga B. Sellera F. P. Cerdeira L. Esposito F. Cardoso B. Fontana H.. (2022). WHO critical priority escherichia coli as one health challenge for a post-pandemic scenario: genomic surveillance and analysis of current trends in Brazil. Microbiol. Spectr. 10, e01256–e01221. doi: 10.1128/spectrum.01256-21, PMID: 35234515 PMC8941879

[B12] Galhano B. S. P. Ferrari R. G. Panzenhagen P. de Jesus A. C. S. Conte-Junior C. A. (2021). Antimicrobial resistance gene detection methods for bacteria in animal-based foods: A brief review of highlights and advantages. Microorganisms 9, 1–15. doi: 10.3390/MICROORGANISMS9050923, PMID: 33925810 PMC8146338

[B13] Gröschel M. I. Owens M. Freschi L. Vargas R. Marin M. G. Phelan J.. (2021). GenTB: A user-friendly genome-based predictor for tuberculosis resistance powered by machine learning. Genome Med. 13, 1–14. doi: 10.1186/S13073-021-00953-4, PMID: 34461978 PMC8407037

[B14] Hauser A. S. Chavali S. Masuho I. Jahn L. J. Martemyanov K. A. Gloriam D. E.. (2018). Pharmacogenomics of GPCR drug targets. Cell 172, 41–54.e19. doi: 10.1016/J.CELL.2017.11.033, PMID: 29249361 PMC5766829

[B15] Hawkey P. M. (2015). Multidrug-resistant Gram-negative bacteria: A product of globalization. J. Hosp. Infection 89, 241–247. doi: 10.1016/j.jhin.2015.01.008, PMID: 25737092

[B16] Jansen K. U. Gruber W. C. Simon R. Wassil J. Anderson A. S. (2021). The impact of human vaccines on bacterial antimicrobial resistance. A review. Environ. Chem. Lett. 19, 4031–4062. doi: 10.1007/S10311-021-01274-Z, PMID: 34602924 PMC8479502

[B17] Kakoullis L. Papachristodoulou E. Chra P. Panos G. (2021). Mechanisms of antibiotic resistance in important gram-positive and gram-negative pathogens and novel antibiotic solutions. Antibiotics 10, 415. doi: 10.3390/ANTIBIOTICS10040415, PMID: 33920199 PMC8069106

[B18] Kim J. Greenberg D. E. Pifer R. Jiang S. Xiao G. Shelburne S. A.. (2020). VAMPr: VAriant Mapping and Prediction of antibiotic resistance via explainable features and machine learning. PLoS Comput. Biol. 16, e1007511. doi: 10.1371/JOURNAL.PCBI.1007511, PMID: 31929521 PMC7015433

[B19] Kwiatek M. Parasion S. Nakonieczna A. (2020). Therapeutic bacteriophages as a rescue treatment for drug-resistant infections – an *in vivo* studies overview. J. Appl. Microbiol. 128, 985–1002. doi: 10.1111/JAM.14535, PMID: 31778593

[B20] Li C. Sutherland D. Hammond S. A. Yang C. Taho F. Bergman L.. (2022). AMPlify: attentive deep learning model for discovery of novel antimicrobial peptides effective against WHO priority pathogens. BMC Genomics 23, 1–15. doi: 10.1186/S12864-022-08310-4, PMID: 35078402 PMC8788131

[B21] Li Y. Xu Z. Han W. Cao H. Umarov R. Yan A.. (2021). HMD-ARG: hierarchical multi-task deep learning for annotating antibiotic resistance genes. Microbiome 9, 1–12. doi: 10.1186/S40168-021-01002-3, PMID: 33557954 PMC7871585

[B22] Lin T.-T. Yang L.-Y. Lu I.-H. Cheng W.-C. Hsu Z.-R. Chen S.-H.. (2021). AI4AMP: an antimicrobial peptide predictor using physicochemical property-based encoding method and deep learning. MSystems 6, 1–11. doi: 10.1128/MSYSTEMS.00299-21, PMID: 34783578 PMC8594441

[B23] Magiorakos A. P. Srinivasan A. Carey R. B. Carmeli Y. Falagas M. E. Giske C. G.. (2012). Multidrug-resistant, extensively drug-resistant and pandrug-resistant bacteria: An international expert proposal for interim standard definitions for acquired resistance. Clin. Microbiol. Infection 18, 268–281. doi: 10.1111/j.1469-0691.2011.03570.x, PMID: 21793988

[B24] Murugaiyan J. Anand Kumar P. Rao G. S. Iskandar K. Hawser S. Hays J. P.. (2022). Progress in alternative strategies to combat antimicrobial resistance: focus on antibiotics. Antibiotics 11, 200. doi: 10.3390/ANTIBIOTICS11020200, PMID: 35203804 PMC8868457

[B25] NCBI (2025). National Database of Antibiotic Resistant Organisms (NDARO) - Pathogen Detection - NCBI. Available online at: https://www.ncbi.nlm.nih.gov/pathogens/antimicrobial-resistance/.

[B26] Ndikuryayo F. Gong X. Y. Hao G. F. Yang W. C. (2025). How artificial intelligence assists in overcoming drug resistance? Medicinal Res. Rev. doi: 10.1002/MED.70002, PMID: 40611789

[B27] Nhu N. T. Q. Young V. B. (2023). The relationship between the microbiome and antimicrobial resistance. Clin. Infect. Dis. 77, S479–S486. doi: 10.1093/CID/CIAD641, PMID: 38051965 PMC11487093

[B28] Ramzan M. Raza A. Nisa Z. U. Abdel-Massih R. M. Al Bakain R. Cabrerizo F. M.. (2024). Detection of antimicrobial resistance (AMR) and antimicrobial susceptibility testing (AST) using advanced spectroscopic techniques: A review. TrAC Trends Analytical Chem. 172, 117562. doi: 10.1016/J.TRAC.2024.117562

[B29] Santos-Júnior C. D. Pan S. Zhao X. M. Coelho L. P. (2020). Macrel: Antimicrobial peptide screening in genomes and metagenomes. PeerJ 8, 1–20. doi: 10.7717/PEERJ.10555, PMID: 33384902 PMC7751412

[B30] Stokes J. M. Yang K. Swanson K. Jin W. Cubillos-Ruiz A. Donghia N. M.. (2020). A deep learning approach to antibiotic discovery. Cell 180, 688–702.e13. doi: 10.1016/j.cell.2020.01.021, PMID: 32084340 PMC8349178

[B31] Tortora G. J. Funke B. R. Case C. L. (2004). Microbiology: An Introduction (San Francisco, California: Pearson/Benjamin Cummings).

[B32] Wang Z. Li S. You R. Zhu S. Zhou X. J. Sun F. (2021). ARG-SHINE: improve antibiotic resistance class prediction by integrating sequence homology, functional information and deep convolutional neural network. NAR Genomics Bioinf. 3, 1–11. doi: 10.1093/NARGAB/LQAB066, PMID: 34377977 PMC8341004

[B33] WHO (2024). WHO Bacterial Priority Pathogens List 2024: bacterial pathogens of public health importance to guide research, development and strategies to prevent and control antimicrobial resistance. (Geneva, Switzerland: World Health Organization).

[B34] Zhou Y. Arribas G. H. Turku A. Jürgenson T. Mkrtchian S. Krebs K.. (2021). Rare genetic variability in human drug target genes modulates drug response and can guide precision medicine. Sci Adv. 7, 1–11. doi: 10.1126/SCIADV.ABI6856, PMID: 34516913 PMC8442892

